# Characteristics associated with optimal blood sugar in individuals living with type 2 diabetes in hard-to-reach rural communities: results of a cross-sectional study in Esmeraldas, Ecuador

**DOI:** 10.1186/s12889-025-22324-z

**Published:** 2025-03-25

**Authors:** Marta Puig-García, Cintia Caicedo-Montaño, Mónica Márquez-Figueroa, Elisa Chilet-Rosell, Blanca Lumbreras, Abraham Beltrán-Pérez, Lucy Anne Parker

**Affiliations:** 1 Department of Public Health, History of Science and Gynaecology, Universidad Miguel Hernández de Elche, Alicante, Spain; 2 CIBER de Epidemiología y Salud Pública (CIBERESP), Madrid, Spain; 3 Centro de Epidemiología Comunitaria y Medicina Tropical (CECOMET), Esmeraldas, Ecuador

**Keywords:** Diabetes Mellitus, Type 2, Rural Population, Glycaemic Control, Noncommunicable Diseases, Cross-Sectional Studies, Ecuador

## Abstract

**Background:**

Type 2 diabetes mellitus (T2DM) is a pressing public health challenge in Latin America, with an increasing prevalence and negative impacts on population health. Achieving optimal blood glucose levels is critical for preventing complications, yet significant socioeconomic inequities persist in disease management and optimal glucose control. We aimed to investigate the patient characteristics associated with optimal fasting capillary glucose in individuals living with T2DM in a hard-to-reach setting in Esmeraldas, Ecuador.

**Methods:**

We carried out a cross-sectional study of individuals with T2DM in the Eloy Alfaro health district of Esmeraldas, using a complex sample design with some limitations. Data collection took place between October 2020 and May 2022 and involved face-to-face interviews to collect sociodemographic and clinical data and a Fasting Capillary Blood Glucose test. Perceived social support was measured with the Multidimensional Scale of Perceived Social Support (MSPSS). We estimated the prevalence of optimal glucose levels according to patient characteristics and calculated odds ratios (OR) with 95% confidence intervals using multivariable logistic regression.

**Results:**

Of the 474 participants surveyed, only 18.1% (86; 95%CI: 14.9–21.9) had optimal fasting capillary glucose levels. In this sample, optimal glucose was nearly four times more frequent among men compared to women (aOR = 3.92, 95%CI: 2.08–7.40, *p* < 0.001). Furthermore, older age (aOR = 1.03, 95%CI: 1.01–1.05, *p* = 0.006), living in an urbanised setting (aOR = 2.04, 95%CI: 1.22–3.40, *p* = 0.006) and unemployment (aOR = 0.48, 95%CI: 0.25–0.94, *p* = 0.031) were also linked to optimal blood glucose levels. While perceived social support in this population was moderate (median = 2.33, on a scale of 1 to 4), high family support appeared to reduce optimal glycaemic levels (aOR = 0.35, 95%CI: 0.18–0.70, *p* = 0.003).

**Conclusions:**

The intricate interplay of factors influencing diabetes management and optimal blood sugar suggests that targeted, context-specific and gender-sensitive public health strategies may be needed to address diabetes disparities in vulnerable populations.

**Supplementary Information:**

The online version contains supplementary material available at 10.1186/s12889-025-22324-z.

## Background

Type 2 diabetes mellitus (T2DM) has emerged as a significant public health challenge in Latin America, as the region has experienced an increase in diabetes prevalence in recent decades, posing a significant threat to the health and well-being of the population [1]. In Ecuador, diabetes was identified in 2022 as the second leading cause of death in the general population with a 5.6% of all deaths in the country [2].

Glycaemic control in patients with diabetes is a pivotal element for averting or delaying complications [3]. To achieve that, patients are advised to adhere to self-management practices, both pharmacological and behavioural [4]. Nonetheless, a common critique is that guidelines supporting these recommendations often overlook the socioeconomic context in which individuals live, particularly how those with socioeconomic challenges may encounter barriers to adhering to self-management practices [5].

There is convincing evidence showing that socioeconomic factors, such as income, education level, ethnicity and employment status influence glycaemic control among people with T2DM [6, 7]. Women, facing biological differences and an elevated risk of low socioeconomic status due to gender roles, tend to have worse glycaemic control than men [8]. Age and disease duration are additional factors that significantly impact diabetes management, underlining the clinical relevance to evaluate these alongside socioeconomic factors [6, 9].

Patients’ social environment, including social support, is not frequently addressed in diabetes management, yet it has consistently been associated with improved glycaemic control [10–12]. Social support can take practical, informational or emotional forms and may come from family, friends, community, etc. helping patients overcome barriers to managing their chronic condition [13, 14].

Understanding the factors influencing glycaemic control is crucial given the rising prevalence of T2DM, especially in challenging and remote settings. Research in these contexts is critical to identify unique determinants and develop tailored interventions that address specific challenges, emphasising the role of social factors in managing non-communicable diseases.

## Methods

### Aim

This study aims to answer the following questions:

• What is the prevalence of optimal fasting capillary glucose levels, and what patient characteristics are associated with these levels in hard-to-reach rural communities and urbanised areas of the Eloy Alfaro health district in Esmeraldas, Ecuador?

### Study design

The present study is a component of the CEAD project – Contextualizing Evidence for Action on Diabetes in low-resource Settings: A Mixed-methods case study in Quito and Esmeraldas, Ecuador. This project employs a mixed-methods approach integrating both quantitative epidemiological analysis and qualitative research to investigate how global recommendations can be tailored into specific, evidence-based actions for diabetes prevention in local contexts. To evaluate the implementation of comprehensive diabetes care in low-resource settings, we conducted a cross-sectional study of individuals living with diabetes in two settings (Quito and Esmeraldas). This study focuses on the Eloy Alfaro health district 08D02 in Esmeraldas, province of Ecuador, where we concurrently conducted a population survey showing a diabetes prevalence of 6.8%, markedly higher among women (10.4%) compared to men (2.0%) [15].

We gathered data on diabetes-related care and clinical outcomes, as well as socio-demographic and clinical patient characteristics using face-to-face patient interviews. Participants provided informed consent to access their clinical records for detailed information on disease management (glycaemic control measurements, frequency of medical check-ups, visits to specialists, complications). Furthermore, following a recommendation from local health promoters we measured glycaemic control with a fasting capillary glucose test at the time of interview. This allowed us to provide immediate feedback to participants on their glucose levels at a time of severely limited access healthcare (COVID-19 pandemic) and considering the scarcity of glucometers and test strips in the area. In this manuscript, we report the fasting capillary glucose result at the time of interview, as it was the most consistently reported glucose outcome for the study participants.

### Setting

The Eloy Alfaro health district is in a densely forested rural area of the northwest coastal region of Esmeraldas province, and has a population of 45,629 (est. 2020) [16]. Different ethnic groups, 85% Afro-Ecuadorian, 10% indigenous Chachi, and 5% mestizo (mixed ethnic heritage), live in small riverside communities along the major rivers, accessible mainly by water, or in the larger villages, Borbón and Valdez, which are referred to here as “urbanised areas”. Economic activities include livestock, agriculture, fishing, and tourism, having the highest unemployment rate of Ecuador (9%) and with 52.3% of the population experiencing unmet basic needs [17].

### Participants

#### Eligibility criteria

We included patients over 18 years old with a medical diagnosis of T2DM prior to January 2019. Pregnant women diagnosed with gestational diabetes, individuals with type 1 diabetes, and those with mental disorders impeding consent were excluded.

#### Study size and sampling strategy

A priori, we had defined a conservative sample size of 576 individuals to estimate the proportion of individuals with poor glycaemic levels of 50% [18]. We initially planned to make a random sample of individuals diagnosed with diabetes who lived in the health district, but after consulting the different databases available for recruitment and considering potential difficulties arising with locating individuals identified in routine databases, we attempted to recruit the entire diabetes population in the study district (official data suggested that there were 587 individuals living with diabetes in the health district).

Given the structure of the district’s health service, patient recruitment varied by rural or urban residence. For patients living in urbanised areas, we used the Ministry of Public Health’s (MSP, from its Spanish acronym) daily automated record of consultations and outpatient care (RDCAA) and the MSPS’s chronic conditions matrix tool. These records include individuals with diabetes attending district hospitals and were expected to capture all urban-based patients with diabetes. In rural communities, where MSP health teams visit infrequently, recruitment relied on the Centre of Community Epidemiology and Tropical Medicine (CECOMET), a non-profit organisation with over 20 years of experience in addressing and monitoring health challenges through community-focused approaches. CECOMET supports MSP through a network of lay health promotors who accompany the health teams and enhance accessibility. The MSP’s record-keeping capacity in rural communities requires paper-based data collection and manual entry into the computer system when returning to the subcentre. Due to significant limitations of this system, CECOMET uses a parallel system to monitor their activities, which was considered to be more complete. As such, we used CECOMET’s internal diabetes database for study recruitment which initially consisted of 530 individuals.

Both databases were refined based on inclusion criteria, removing duplicates, deceased individuals, and confirmed people without type 2 diabetes. Each person received a unique code, comprising their community acronym and a sequential number, to ensure confidentiality.

Data collection was organised in two phases: 1st) October 2020 to August 2021 recruitment in rural communities was aligned with the CECOMET’s programmatic visits to selected rural communities and parallel recruitment in the urbanised settings; 2nd) December 2021 to May 2022, we visited new rural communities with the specific purpose of study recruitment, prioritising those with a higher number of people with diabetes and easier accessibility.

**Fig. 1:**
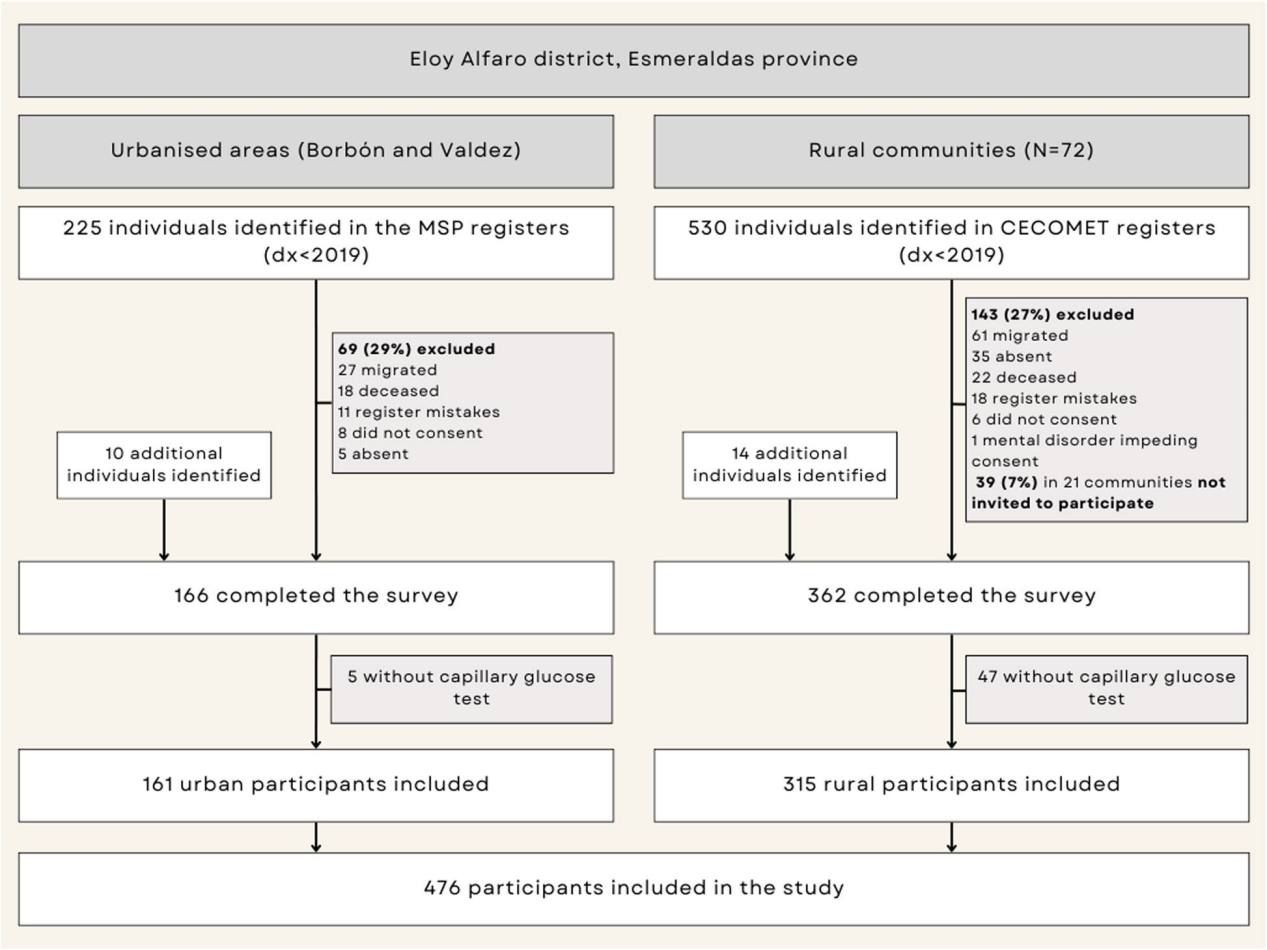
Study recruitment flow diagram

During recruitment, some additional patients were identified (e.g. patients with diabetes who met the inclusion criteria but who did not appear in the databases who were detected during medical record searches or known by local health promoters). On the other hand, a significant proportion of individuals listed in the databases could not be reached at the time of data collection because they were either temporally absent (5.3%) (e.g. due to work or health reasons) or had migrated to other communities (11.7%), perhaps influenced by the pandemic period (Fig. 1). Health promoters were informed in advance of the study team’s visit to facilitate patient mobilisation and maximise participation. Additionally, 52 individuals did not undergo capillary blood glucose testing because they were not in a fasting state at the time of measurement. This was more common among women (42 individuals, 16.2%) than men (10 individuals, 9.6%).

### Data collection

The survey team included 2 nursing assistants and 7 healthcare professionals (6 nurses and an obstetrician) familiar with the community. Two CECOMET public health nurse coordinators provided technical and logistical support. Local health promoters and nursing assistants actively participated in community interviews, assisting with participant recruitment, house location, and fasting instructions for glucose testing. At least one member of the team was able to translate questions verbally to Cha’palaa (language spoken by the indigenous population in the area) when needed.

Local health promoters located individuals who had been identified for inclusion in the study. They informed them of the study’s objectives, and interview date and instructed them to attend the interviews without having eaten anything from 9 pm the night before. On the interview day, participants met with the study team in an agreed location (e.g. at the health promoter’s house) early in the morning where they had a fasting capillary glucose test and then, after having breakfast, the survey team visited their homes individually to complete the interview. All fasting capillary glucose tests were performed using the same Accu-Chek Instant© device (Roche) to standardise the measurements and minimise variability. The questionnaire used for the interviews was specifically designed for this study, combining elements based on different instruments (Supplementary File 1). We collected socioeconomic and demographic data following the structure of the WHO STEPS survey. Clinical data, health services, and access to treatment were gathered based on recommendations from the National Clinical Practice Guideline [19], with some ad hoc questions related to the impact of the COVID-19 pandemic. Additionally, we assessed Health-Related Quality of Life using the EuroQol-5D (EQ-5D) instrument and measured perceived social support with the Multidimensional Scale of Perceived Social Support (MSPSS) [20]. For this study analysis we used socioeconomic data and the MSPSS instrument, which measures three sources of social support: significant other, family, and friends using 12 questions. We chose the adaptation by Arechabala and Miranda, originally designed for Chilean elderly patients with hypertension [21], which uses a Likert-type scale ranging from 1 (almost never) to 4 (always or almost always) rather than the original 1 to 7. This version has also been applied to Chilean patients with T2DM [22]. We conducted a pre-pilot study with patients from the area, during which no issues were identified regarding the linguistic comprehension of the survey tool. As psychometric validation had not yet been conducted in an Ecuadorian population, we used this sample to evaluate the internal consistency of the MSPSS in this population using Cronbach’s alpha and performed a Confirmatory Factor Analysis (CFA). Survey data were recorded on digital tablets (Samsung Galaxy Tab AT290) using the KoboCollect program (version 2.4).

### Variables

The main outcome variable of this study is Fasting Capillary Blood Glucose (FCBG), defined as optimal when the FCBG levels were between 80–130 mg/dL according to ADA guidelines [4] and as suboptimal when the levels were over 130 mg/dL. Two individuals had a FCBG below 80 mg/dL which should also be considered suboptimal (hypoglycaemic). They are reported in the descriptive table as a separate category but excluded from the rest of the analysis.

Age was used as a continuous variable. Self-reported ethnicity was simplified to Afro-Ecuadorian, Mestizo and Indigenous. Three categories were used to categorise education: no formal schooling, primary school completion, and secondary school completion or above. Marital status was classified into two categories: partnered (married or free union) or unpartnered (single, separated or widow). Employment status was dichotomised into formal employment (including self-employed, private sector employee or government employee) or not in formal employment (homemakers, students, unemployed or retired). The item for estimated household earnings was divided according to the median ($0 to $100 and over $100). The duration of T2DM was calculated by subtracting the self-reported diagnosis date from the interview date and used as a continuous variable. If an individual was unable to recall the diagnosis date, we used the date found in the participant’s medical history. Access to free T2DM treatment was based in response to the question “Does the subcentre or hospital provide you with the prescribed medications when you go for a check-up?”. To avoid categories with too few responses, we categorised the answers into ‘always’ and ‘sometimes/never.’

We described the perceived social support of the participants using the mean scale score of the MSPSS instrument [20], overall and in each of the three subdomains (family, friends, and significant others), each ranging between 1 (low perceived support) and 4 points (high perceived support). In multivariable analysis, we dichotomised each support variable in low (mean MSPSS scale score < 2.5) and high support (mean MSPSS scale score ≥ 2.5). For participants with no more than one unanswered question per subdomain, the mean was recalculated accounting for the missing value. When there was more than one unanswered question in a subdomain, all social support variables were treated as missing values and excluded from the analysis.

### Data analysis

We used Stata version 15.0 (StataCorp, College Station, TX, USA) for all statistical analyses. We described the prevalence of our main outcome variable with 95% confidence intervals, disaggregated by sex. We evaluated the normality of the data with the Shapiro–Wilk test. We compared the sociodemographic, clinical, and social support features using proportions and Fisher’s exact test for categorical variables, the mean and Student’s t-test for continuous variables with a normal distribution and the median and interquartile range (IQR) and Kruskal–Wallis test for variables with a non-normal distribution. We used logistic regression to estimate odds ratios (ORs) with 95% confidence intervals. We conducted a multivariable logistic regression analysis to identify significant predictors of optimal fasting glucose. Variables were entered into the model if they met a *p*-value threshold of < 0.10 for inclusion and were retained if they maintained a *p*-value of < 0.05. This analysis was performed using the stepwise command in Stata. As a sensitivity analysis, we also restricted the sample to individuals over 40 years old, and to facilitate gender analysis in interpreting the results we explored other models stratified by sex using the backward stepwise elimination method. Missing data were excluded from the logistic regression analysis. We assessed multicollinearity using the correlation matrix and the Variance Inflation Factor (VIF).

## Results

Five hundred twenty eight individuals with T2DM were interviewed, of whom 476 (90.2%) had a result of fasting capillary glucose and therefore were included in this study (Fig. 1). Most participants were women (*N* = 334, 70.2%) (Table 1). The mean age was 60 ± 13 years, similar for both sexes (range 22–91 years). The distribution of ethnic groups shows a majority of Afro-Ecuadorians (65.4%), followed by Mestizo (29.5%) and Indigenous (5.1%) with 66.2% living in rural communities (*N* = 315). The median duration of diabetes was 7 years with an IQR of 5–11 years and ranging from 2 to 54 years (not shown in table), without differences by sex.

**Table 1:** Description of the sociodemographic, clinical and social support characteristics (*N* = 476)

Characteristics	**Women *n* (%)**	**Men *n* (%)**	**Total *n* (%)**	*p*-value**
**Age** in years (mean ± SD)	(59 ± 13)	(62 ± 13)	(60 ± 13)	0.054
**Ethnic group**^**a**^				0.648
Afro	219 (65.6)	91 (65.0)	310 (65.4)	
Mestizo	100 (29.9)	40 (28.6)	140 (29.5)	
Indigenous	15 (4.5)	9 (6.4)	24 (5.1)	
**Setting**				0.751
Rural	219 (65.6)	96 (67.6)	315 (66.2)	
Urbanised	115 (34.4)	46 (32.4)	161 (33.8)	
**Education level**				**0.014**
No formal schooling	65 (19.5)	23 (16.2)	88 (18.5)	
Primary school	205 (61.4)	74 (52.1)	279 (58.6)	
Secondary or higher	64 (19.2)	45 (31.7)	109 (22.9)	
**Marital status**				0.078
Unpartnered	105 (31.4)	33 (23.2)	138 (29.0)	
Partnered	229 (68.6)	109 (76.8)	338 (71.0)	
**Employment status**				** < 0.001**
Unemployed	275 (82.3)	38 (26.8)	313 (65.8)	
Employed	59 (17.7)	104 (73.2)	163 (34.2)	
**Household earnings**^b^				** < 0.001**
$100 or less	188 (61.0)	51 (39.5)	239 (54.7)	
More than $100	120 (39.0)	78 (60.5)	198 (45.3)	
**Capillary glucose result**				** < 0.001**
Hypoglycaemia (< 80 mg/dL)	1 (0.3)	1 (0.7)	2 (0.4)	
Optimal (80–130 mg/dL)	46 (13.8)	40 (28.2)	86 (18.1)	
Hyperglycaemia (> 130 mg/dL)	287 (85.9)	101 (71.1)	388 (81.5)	
**T2DM duration,** mean in years (IQR)^c^	7 (5–11)	7 (5–11)	7 (5–11)	0.867
**Free T2DM treatment provided by the health centre** (self-reported)^**d**^				0.475
Sometimes/never	179 (54.6)	80 (58.4)	259 (55.7)	
Always	149 (45.4)	57 (41.6)	206 (44.3)	
**Total social support**^e^				0.541
Low	207 (62.7)	87 (61.7)	294 (62.4)	
High	123 (37.3)	54 (38.3)	177 (37.6)	
**Family support**^e^				0.347
Low	42 (12.7)	13 (9.2)	55 (11.7)	
High	288 (87.3)	128 (90.8)	416 (88.3)	
**Friend support**^e^				0.499
Low	244 (73.9)	100 (70.9)	344 (73.0)	
High	86 (26.1)	41 (29.1)	127 (27.0)	
**Other significant support**^e^				1.000
Low	226 (68.5)	97 (68.8)	323 (68.6)	
High	104 (31.5)	44 (31.2)	148 (31.4)	
**Total**	334 (100%)	142 (100%)	476 (100%)	

^a^2 missing

^b^39 not reported

^c^7 missing

^d^11 missing

^e^5 missing social support (MSPSS scale)

^**^p-value for the Fisher’s exact test comparing men and women

The sociodemographic characteristics varied between men and women. While more than half of the participants had reached primary schooling (*N* = 279), men showed a significantly higher education level. Men also reported having a partner more often than women (109 men, 76.8% versus 229 women, 68.6%, *p* = 0.078). Women were more likely to be unemployed (*N* = 275, 82.3%) compared to men (*N* = 38, 26.8%, *p* =  < 0.001) Household earnings further emphasise disparities, as 61% of women (*N* = 188) declared earning $100 or less per month, compared to 40% of men.

Regarding perceived social support, the psychometric validation of the MSPSS showed excellent internal consistency with a Cronbach’s alpha of 0.91 (95%CI: 0.91–0.92), and a robust factor structure confirmed by CFA indices: Tucker–Lewis Index of 0.998, Root Mean Square Error of Approximation of 0.999, and Root Mean Square Error of Approximation of 0.119 (Full details of the analysis are provided in the Supplementary File 2). Participants who had answered the MSPSS (*N* = 471) reported a median score of 2.33 (IQR: 1.83–2.83) on the MSPSS, indicating a moderate level of overall perceived social support, with 62.4% (*N* = 294) experiencing low social support in the dichotomised variable. However, further exploration of the subdomains of social support showed a disparity between the perceived family support, with higher scores (median: 3.5, IQR: 3–4) compared to friend support (median: 2, IQR: 1.25–2.5) and other significant support (median: 2, IQR: 1–3). We did not detect differences in social support between men and women (Table 1).

### Optimal capillary glucose levels and associated factors

Analysis of the fasting capillary glucose tests showed a median value of 210.5 mg/dL (IQR: 144–285), with the range of values ​between 62 and 571 mg/dL. Remarkably, only 86 individuals, representing 18.1% of the sample (95%CI: 14.9–21.9), had optimal glucose levels within the optimal range of 80–130 mg/dL. Among the other 390 individuals, 388 presented suboptimal results above 130 mg/dL, indicating an 81.9% (95%CI: 78.2–85.1) of hyperglycaemia in the population studied.

Notably, the prevalence of an optimal outcome was significantly higher in men than in women (40 men, 28.4% (95%CI: 21.5–36.4); compared to 46 women, 13.8% (95%CI: 10.5–18.0)) (Table 2). Individuals with optimal glycaemia had a higher mean age (64 ± 15 years) compared to those with suboptimal glycaemia (60 ± 13 years, *p* = 0.001). However, when disaggregated by sex, this pattern was only observed among men (Table 2). Individuals living in urbanised areas had a higher proportion of an optimal result of FCBG (23.6%) than their rural counterparts (15.3%, *p* = 0.032). When disaggregating by sex this association was only observed among women (Table 2).

**Table 2:** Prevalence of optimal Fasting Capillary Blood Glucose result according to sociodemographic and clinical characteristics disaggregated by sex (*N* = 474)

	**Total population**			**Women**			**Men**		
**Characteristics**	***N* (%) optimal**	**Total**	*p*-value**	***N* (%) optimal**	**Total**	*p*-value**	***N* (%) optimal**	**Total**	*p*-value**
**Sex**			** < 0.001**						
Women	46 (13.8)	333		-	-	-	-	-	-
Men	40 (28.4)	141		-	-	-	-	-	-
**Age** (mean ± SD)	(64 ± 15)	(60 ± 13)	** < 0.001**	(60 ± 16)	(59 ± 13)	0.459	(68 ± 13)	(62 ± 13)	** < 0.001**
**Ethnic group**^**a**^			0.453			0.576			0.781
Mestizo	22 (15.7%)	140		12 (12.0%)	100		10 (25.0%)	40	
Afro	58 (18.8%)	308		31 (14.2%)	218		27 (30.0%)	90	
Indigenous	6 (25.0%)	24		3 (20.0%)	15		3 (33.3%)	9	
**Setting**			**0.032**			**0.011**			0.696
Rural	48 (15.3%)	313		22 (10.1%)	218		26 (27.4%)	95	
Urbanised	38 (23.6%)	161		24 (20.9%)	115		14 (30.4%)	46	
**Education level**			0.144			0.385			**0.032**
No formal schooling	22 (25.3%)	87		12 (18.8%)	64		10 (43.5%)	23	
Primary school	48 (17.3%)	278		25 (12.2%)	205		23 (31.5%)	73	
Secondary or higher	16 (14.7%)	109		9 (14.1%)	64		7 (15.6%)	45	
**Marital status**			0.429			0.864			0.117
Unpartnered	28 (20.6%)	136		15 (14.4%)	104		13 (40.6%)	32	
Partnered	58 (17.2%)	338		31 (13.5%)	229		27 (24.8%)	109	
**Employment status**			1.000			1.000			**0.001**
Unemployed	57 (18.3%)	312		38 (13.9%)	274		19 (50.0%)	38	
Employed	29 (17.9%)	162		8 (13.6%)	59		21 (20.4%)	103	
**Household earnings**^b^			0.214			0.184			0.158
$100 or less	49 (20.7%)	237		31 (16.6%)	187		18 (36.0%)	50	
More than $100	31 (15.7%)	198	0.942	13 (10.8%)	120		18 (23.1%)	78	
**T2DM duration,** mean in years (IQR)^c^	7 (5–13)	7 (5–11)	0.938	7 (5–12)	7 (5–11)	0.791	6 (5–15)	8 (5–11)	0.908
**Free T2DM treatment provided by the health centre** (self-reported)^d^			0.621			1.000			0.337
Sometimes/never	42 (16.3)	257		23 (12.9)	178		19 (24.1)	79	
Always	38 (18.5)	206		20 (13.4)	149		28 (34.6)	57	
**Social support**^e^			0.536			0.869			0.443
Low	56 (19.1%)	293		29 (14.0%)	207		27 (31.4%)	86	
High	29 (16.5%)	176		16 (13.0%)	122		13 (24.1%)	54	
**Family support**^e^			**0.025**			**0.053**			0.101
Low	16 (29.6%)	54		10 (23.8%)	42		6 (50.0%)	12	
High	69 (16.6%)	415		35 (12.2%)	287		34 (26.6%)	128	
**Friend support**^e^			1.000			0.367			0.309
Low	62 (18.1)	343		31 (12.7%)	244		31 (31.3%)	99	
High	23 (18.3)	126		14 (16.5%)	85		9 (22.0%)	41	
**Other significant support**^e^			0.700			0.494			0.844
Low	60 (18.7)	321		33 (14.7%)	225		27 (28.1%)	96	
High	25 (16.9)	148		12 (11.5%)	104		13 (29.6%)	44	
**TOTAL**	**86 (18.1%)**	**474**		**46 (13.8%)**	**333**		**40 (28.4%)**	**141**	

^a^2 missing

^b^39 not reported

^c^7 missing

^d^11 missing

^e^5 missing social support (MSPSS scale)

^**^p value marked in bold when < 0.1 (t-student for age and Fisher’s exact test for the rest)

The multivariable regression analysis revealed that men were nearly four times more likely than women to achieve optimal glycaemic control, even after adjusting for other covariates in the model (aOR = 3.92, 95%CI: 2.08–7.40, p < 0.001) (Table 3). Each additional year of age was associated with a 3% increase in the odds of achieving optimal blood glucose levels (aOR = 1.03, 95%CI: 1.01–1.05, *p* = 0.006). Individuals residing in urbanised areas also had significantly higher odds of an optimal glycaemic result compared to those in rural areas (aOR = 2.04, 95%CI: 1.22–3.40, *p* = 0.006). Employment status was inversely associated with glycaemic control, with employed participants having significantly lower odds of achieving optimal fasting glucose levels compared to unemployed individuals (aOR = 0.48, 95%CI: 0.25–0.94, *p* = 0.031). Finally, higher perceptions of family support were associated with lower odds of achieving an optimal glycaemic result. Participants reporting high family support were 65% less likely to achieve optimal glycemia compared to those with low family support (aOR = 0.35, 95%CI: 0.18–0.70, *p* = 0.003). The multicollinearity analysis indicated minimal correlation among the variables. The highest correlation was 0.5153 between sex and employment, with VIF values of 1.71 for employment and 1.44 for sex.

**Table 3:** Patient characteristics associated with an optimal Fasting Capillary Blood Glucose result (*N* = 474)

	**Total**					
Characteristics	**OR**	**95% CI**	***p*-value**	adjusted OR**	**95% CI**	***p*-value**
**Sex**						
Women	1			1		
Men	2.47	1.53–4.00	** < 0.001**	3.92	2.08–7.40	** < 0.001**
**Age**	1.03	1.01–1.05	**0.001**	1.03	1.01–1.05	**0.006**
**Ethnic group**^**a**^						
Mestizo	1					
Afro	1.24	0.73–2.13	0.425			
Indigenous	1.79	0.64–5.00	0.269			
**Setting**						
Rural	1			1		
Urban	1.71	1.06–2.75	**0.028**	2.04	1.22–3.40	**0.006**
**Education level**						
No formal schooling	1					
Primary school	0.62	0.35–1.10	0.099			
Secondary or higher	0.51	0.25–1.04	0.065			
**Marital status**						
Unpartnered	1					
Partnered	0.80	0.48–1.32	0.382			
**Employment status**						
Unemployed	1			1		
Employed	0.98	0.60–1.60	0.921	0.48	0.25–0.94	**0.031**
**Household earnings**^b^						
$100 or less	1					
More than $100	0.71	0.43–1.17	0.180			
**T2DM duration,** mean in years (IQR)^c^	1.02	0.98–1.05	0.388			
**Free T2DM treatment provided by the health centre (**self-reported)^d^						
Never/sometimes	1					
Always	1.16	0.71–1.88	0.552			
**Social support**^e^						
Low	1					
High	0.84	0.51–1.37	0.474			
**Family support**^e^						
Low	1			1		
High	0.47	0.25–0.90	**0.022**	0.35	0.18–0.70	**0.003**
**Friend support**^e^						
Low	1					
High	1.01	0.60–1.72	0.965			
**Other significant support**^e^						
Low	1					
High	0.88	0.53–1.48	0.638			

^a^2 missing

^b^39 not reported

^c^7 missing

^d^11 missing

^§^5 missing social support (MSPSS scale)

^**^OR adjusted for variables (sex, age, setting, employment and family support) with sample size of 469 due to 5 missing values in family support

The multivariable regression sub-analysis for women revealed that those residing in urbanised areas had higher odds of achieving optimal glycaemic levels (aOR = 2.33, 95%CI: 1.23–4.42, *p* = 0.010), after adjusting for family support (Supplementary File 3, Table S1). Women with higher perceptions of family support showed a non-significant trend toward lower odds of optimal glycaemia (aOR = 0.48, 95%CI: 0.22–1.08, *p* = 0.077). Among men, age, education and employment status appeared to be significant factors. Men with higher education levels had a lower prevalence of optimal glycaemic levels (15.6%) compared to those with no formal education (43.5%) and primary school (31.5%, *p* = 0.032). Similarly, optimal glycaemia was less frequent among employed men (20.4%) than among unemployed men (50.0%, *p*: 0.001). However, these associations lost statistical significance when adjusted for age (Supplementary File 3, Table S2). Notably, older men had significantly higher odds of achieving optimal fasting glycaemia (aOR = 1.04, 95%CI: 1.00–1.08, *p* = 0.045), while unemployment showed a non-significant trend toward improved glycaemic outcomes (aOR-employed = 0.46, 95%CI: 0.18–1.15, *p* = 0.098).

The sensitivity analysis conducted on individuals aged 40 or older continued to show that men were more likely to achieve optimal glycaemia compared to women (OR = 2.91, 95%CI: 1.73–4.89, p < 0.001) (Supplementary File 3, Table S3). Age also played a significant role, with each additional year increasing the odds of achieving optimal glycaemia by 5% (OR = 1.05, 95%CI: 1.02–1.07, p < 0.001). As previously seen, higher family support was associated with lower odds of achieving optimal glycaemic levels, with individuals reporting high family support being 60% less likely to achieve optimal glycaemia compared to those with low family support (OR = 0.40, 95%CI: 0.19–0.86, *p* = 0.019) (Supplementary File 3, Table S4).

## Discussion

Our study found that nearly four-fifths of our sample of individuals with T2DM in the Eloy Alfaro District of Esmeraldas, Ecuador exhibited suboptimal fasting capillary glucose levels. This result is consistent with findings from other studies across Latin America reporting high rates of uncontrolled T2DM [8, 23–25]. This alarming prevalence highlights the urgent need for context-specific interventions that address challenges faced by individuals living in these settings for optimal T2DM management. Such measures are essential to prevent complications related to hyperglycaemia and to improve the quality of life for individuals living with diabetes. In this study, optimal glycaemic levels were associated with certain patient characteristics, including being male, older age, living in urban areas, unemployment, and lower family support. These findings point to the complex interplay of demographic and social factors in diabetes management.

The study area, characterised by its challenging accessibility, is situated within an obesogenic environment marked by the high availability of unhealthy foods, alcohol, and tobacco, alongside limited access to medical care and essential diabetes self-management resources, such as monitoring strips and medication [26, 27]. In addition to these contextual factors, it is crucial to acknowledge that the study was conducted during a period dominated by the COVID-19 pandemic, impacting healthcare access, the ability to work and generate household income, and the nature of social relationships due to the implemented preventive measures [28, 29]. These circumstances may have posed additional challenges to achieving optimal glycaemic levels, further complicating the analysis of associated characteristics.

Our results reveal notable gender-based disparities in fasting glucose levels with men having optimal glycemia at a frequency four times more frequently than women. Women typically experience poorer glycaemic outcomes due to social disadvantages, psychosocial challenges, and physiological factors [30, 31]. These barriers are further exacerbated in rural settings, where deeply ingrained traditional gender roles significantly influence diabetes management [32, 33]. For example, in these settings, where fieldwork and agricultural activities are prevalent, men often have physically demanding jobs that appear to contribute more to blood glucose regulation than the typically more sedentary activities performed by women [26].

In line with the results of other studies, advancing age was associated with better glycaemic levels, often due to improved disease management [30, 34, 35]. While employment is usually related to better glycaemic control [36], the demands of being employed, such as lack of time or permission to attend medical appointments, are also recognised as barriers to diabetes management in Latin America [5]. This could explain why, in our study, employed individuals had poorer glycaemic levels. Interestingly, both older age and unemployment remained significantly associated with optimal glycaemia in our subgroup analysis of men, but were not detected as predictors of optimal glycaemic levels among women. It is possible that despite no longer being employed in manual labour, older men remain active by going to their “finca” (plot of land used for subsistence farming), benefiting from more available time [26]. In contrast, women, as a result of their lifelong dedication to reproductive work, continue to be the family’s primary caregivers and often put family and caregiving before their own health, regardless of age [37].

Our study identified living in an urbanised setting as a factor positively associated with optimal glycemia. This finding is consistent with Flood et al., where individuals with diagnosed diabetes in rural areas had a 14% lower relative risk of achieving glycaemic control compared to their urban counterparts [38]. It is possible that any social disadvantages may be partially mitigated by the easier access to medication and care in urbanised areas, where proximity to pharmacies and health centres can improve diabetes management. The significance of the setting appeared to be particularly important for women, which perhaps reflects the entrenched gender roles in these isolated rural communities.

Our study did not identify an association between disease duration and optimal control. Indeed, other studies show conflicting results with the direction of association here [6, 36]. On one hand, increased disease duration has been linked to improved self-management practices, as individuals gain experience with their condition; on the other hand, it is associated with higher rates of complications and deteriorating health outcomes over time [23, 39]. Finally, when we restricted our analysis to individuals over 40 years old, living in an urban setting and employment status were no longer identified as characteristics associated with optimal glycemia. It is possible that this is due to reduced sample size but it could also suggest these factors are more relevant for younger people.

Contrary to numerous studies [40–43], our findings reveals an unexpected potential link between higher levels of family social support, particularly among women, and suboptimal glycaemic levels. This association may be explained by the limitations of the MSPSS tool used in our study, as it only measures perceived social support from different sources, without distinguishing between different types of support (instrumental, emotional, and informational). While both objective (tangible) and subjective (perceived) support influence well-being, a more comprehensive analysis in this challenging environment, characterised by increased management barriers, would have benefited from exploring whether perceived support translates into tangible support, actually helping to obtain resources for diabetes treatment as done in other rural studies [43, 44]. These results suggest that the role of social support may be compromised in a rural, difficult-to-access setting where needs are largely unmet and social support dynamics are primarily family-focused.

The study’s primary limitation lies in the representativeness of its sample. Although we initially intended to capture the entire eligible population, practical fieldwork constraints in this region, characterised by dense forests, isolated villages, and inadequate data records (a prevalent issue in low- and middle-income countries), required adjustments to our methodology. For efficiency reasons, some isolated communities with a low number of diabetes cases were not visited. This affected 39 individuals, 31 of them women, in 21 communities, representing 5.2% of those in official registers. We might consider that diabetes control is even more challenging in these isolated communities, and therefore, the exclusion of these communities may have led to an underestimation of the prevalence of optimal fasting capillary glucose levels.

Even in the communities that were visited, we were only able to recruit individuals who could be located. Overall, 6.6% of individuals in rural areas and 2.2% in urban areas were absent at the time of data collection, while 11.5% of those listed in rural areas and 12.0% in urban areas had migrated. Additionally, we identified previously unregistered individuals with diabetes in both urban and rural areas. These patterns suggest that our sample may be biased towards individuals with greater access to healthcare services, potentially leading to an overestimation of optimal glycaemic results. Moreover, individuals who had died, possibly due to diabetes-related complications, were excluded from the study, further contributing to potential bias. On the other hand, it is possible that those excluded because of migration represent a healthier segment of the population, as suggested by the "healthy migrant effect” [45]. This could have led to an overrepresentation of individuals with poorer health outcomes in our sample.

Finally, regarding demographic differences between included and excluded participants, we found that the proportion of women and men was similar in both groups, with no notable differences between urban and rural areas. While this suggests that exclusion did not introduce systematic bias in terms of sex or geographical distribution, we cannot rule out differences in other demographic or socioeconomic characteristics, which were not available for comparison.

While selection bias may have influenced estimates of glycaemic outcomes, we are confident that the key associations identified in this study remain robust. Some of the observed effect sizes, such as those related to gender, were large, making them less susceptible to minor variations introduced by selection bias. Furthermore, many of our findings align with those reported in previous studies, as mentioned.

Another limitation of the study is the use of a single capillary blood glucose measurement, which reflects a single point in time only and could miss other factors altering glucose concentrations [46], possibly leading to an over or underestimation of the prevalence of suboptimal glycaemia. Nevertheless, this test was used for all participants in the same conditions meaning we can rely on the findings our analysis regarding the patient characteristics associated with optimal capillary blood glucose, as any measurement bias introduced is non-differential and would bias our OR towards the null, meaning the true OR is equal to or greater in magnitude.

The cross-sectional design of this study represents a significant limitation, as it prevents the determination of causal relationships between the factors analysed and glycaemic control. While associations were identified, there is a clear need for longitudinal studies to clarify the temporal dynamics between these variables. Finally, the limited sample size, particularly in the male subgroup, constrained our ability to conduct robust sex-stratified analyses. This reduced statistical power may have hindered the detection of true differences in optimal glycemia between subgroups. Consequently, we chose to present the analysis for the total population in the main manuscript while including the subgroup analysis findings in the supplementary materials.

Regarding the external validity of our findings, it is important to acknowledge that this study was conducted in a very specific setting of Ecuador, which may limit its generalisability to other contexts. However, isolated, culturally diverse communities with limited healthcare access, similar to those studied here, are present not only in other parts of Ecuador and Latin America but also in various regions worldwide. The alignment of our findings with other studies enhances their credibility and suggests they may be relevant to other rural settings, particularly in low- and middle-income countries. The limited availability of studies examining the psychosocial determinants of non-communicable diseases in rural settings, particularly in low- and middle-income countries, highlights the critical need for further research in this area.

## Conclusions

Our study in Esmeraldas reveals a concerning prevalence of suboptimal glycaemic levels, with only 18.1% of the participants having glycemia within the optimal range. While the exact figure should be interpreted with caution given the recruitment challenges, pandemic context of the fieldwork and the reliance on a single FCGB measure, the findings revealed marked gender disparities, with women facing significantly lower chances of maintaining optimal blood sugar. Other key social determinants influencing optimal glycaemia in this setting include employment as a potential barrier, older age as a protective factor, urban residency being advantageous compared to living in the isolated rural communities, and higher perceived family support unexpectedly showing a negative association. These results underscore the complex interactions among health system, social, and environmental factors in shaping glycaemic outcomes. Policymakers should urge the implementation of gender-specific public health strategies and tailored health programs addressing contextual factors that contribute to better disease management, thereby mitigating disparities and improving overall health outcomes.

## Supplementary Information

Supplementary Material 1.

Supplementary Material 2.

Supplementary Material 3.

## Data Availability

The datasets generated and analysed during the current study are available in the Zenodo repository, with the DOI: 10.5281/zenodo.11105070.

## Abbreviations

T2DM: Type 2 Diabetes Mellitus
CECOMET: Centre of Community Epidemiology and Tropical Medicine
MSP: Public Health Ministry (Ministerio de Salud Pública in Spanish)
RDCAA: Record of Consultations and Outpatient Care (Registro Diario Automatizado de Consultas y Atenciones Ambulatorias in Spanish)
MSPSS: Multidimensional Scale of Perceived Social Support
FCBG: Fasting Capillary Blood Glucose
ADA: American Diabetes Association
IQR: Interquartile range
OR: Odds Ratio
aOR: Adjusted Odds Ratio
CFA: Confirmatory Factor Analysis
