# Supplementary material for: Characteristics associated with optimal blood sugar in individuals living with type 2 diabetes in hard-to-reach rural communities: results of a cross-sectional study in Esmeraldas, Ecuador
Source: BMC Public Health. 2025 Mar 25;25:1133. doi: 10.1186/s12889-025-22324-z (PMC11934518; doi:10.1186/s12889-025-22324-z)
Supplement: Supplementary file 1 — Supplementary Material 1. [file 12889_2025_22324_MOESM1_ESM.docx]

| **QUESTIONNAIRE (English version): Project CEAD Part-II, Strengthening the Health System - Cohort study Esmeraldas** |
| --- |
| **Socio-demographic data** |
| **1. What gender do you identify with?**   - Male - Female - Other   - Specify gender: ____ |
| **2. Do you remember your exact date of birth?**   - Yes [go to Q3] - No [skip to Q4] |
| **3. What is your date of birth?:** yyyy-mm-dd |
| **4. How old are you?**: _____ (If unknown: 777 / If refused to answer: 999) |
| **5. What is your country of birth?**   - Ecuador [go to Q6] - Other [go to Q7] |
| **6. Which province are you from?:** ____ |
| **7. Specify the country of birth if not Ecuador:** ____ |
| **8. Have you had to leave your community for work reasons?**   - Yes - No - Refused to answer |
| **9. How do you identify yourself according to your race (ethnicity) and customs?**   - Indigenous - Afro-descendant - Black - Mulatto - Montubio - Mestizo - White - Other - Refused to answer |
| **10. Language of the interview**   - Spanish - Other: _____ |
| **11. What is your marital status?**   - Single - Married - Living with partner - Widowed - Separated or divorced - Refused to answer |
| **12. What is the highest level of education you have completed?**   - Illiterate - Literacy centre - Primary school - Basic education - Secondary school/High school - Non-university higher education (technical-technological) - University higher education - Postgraduate - Don't know/No answer |
| **13. What was your occupation in 2019?**   - Government employee - Private sector employee - Self-employed - Volunteer worker - Student - Homemaker - Retired - Unemployed (able to work) - Unemployed (unable to work) - Refused to answer |
| **14. Has your employment situation changed in the last 12 months?**   - Yes - No [skip to Q16] - No answer [skip to Q16] |
| **15. What has been your occupation in the last 12 months?**   - Government employee - Private sector employee - Self-employed - Volunteer worker - Student - Homemaker - Retired - Unemployed (able to work) - Unemployed (unable to work) - Refused to answer |
| **16. How much income does your family receive in a month? (What was your household’s average income over the last year?):** ____ (If unknown: 7 [go to Q17] / If refused to answer: 9 [go to Q18]) |
| **17. If you don't know the exact amount, could you give us an estimate of your family's monthly income if I read some options aloud? The amount is:**   - From $0 to $375 - From $376 to $724 - From $725 to $1087 - From $1088 to $1449 - More than $1449 - Don't know - Refused to answer |
| **18. Compared to your situation before the COVID crisis, how do you assess your condition today?** (Before the COVID crisis refers to the year 2019 onwards. Condition refers to economic, family, health, or other characteristics that the patient considers)   - Very deteriorated - Slightly deteriorated - Unchanged - Slightly improved - Greatly improved - No answer [skip to Q20] |
| **19. Why?:** ________  Examples: Employment: lost job, salary reduced or increased... Family: lost a family member, separated... Health: suffered from COVID or another illness, diabetes worsened or improved... |
| **Time to reach the health centre using that mode of transportation** |
| **20. When you usually go to the health centre, what means of transportation do you use?:** _____ |
| **21. Approximately, how long does it take to reach the health centre using that mode of transportation?** (If unknown: 777 / If refused to answer: 999)  Hours: ____  Minutes: ____ |
| **Clinical data** |
| **22. Do you remember when you were diagnosed with diabetes?** Diabetes diagnosis date   - Yes - No [skip to Q24] |
| **23. Year of diabetes diagnosis:** yyyy |
| **24. Or how many years have you had diabetes?:** ______ In case they don’t remember, indicate 777 |
| **25. Do you visit the doctor for diabetes check-ups?**   - Yes - No [skip to Q27] - No answer [skip to Q27] |
| **26. How often do you visit the doctor to control your diabetes?**   - Monthly - Every 3 months - Every 6 months - Annually - Longer than a year - Only visit the doctor when feeling unwell - No answer - Other   - Other frequency of medical visits: _____ |
| **27. Do you monitor your blood sugar levels?** Whether through lab tests or tests at the health subcentre   - Yes - No [skip to Q30] - No answer [skip to Q30] |
| **28. When you monitor your blood sugar, how do you do it?**   - Fasting blood sample for the lab - Test strip - Both - Other   - Other type of blood sugar monitoring: ____ |
| **29. How often do you check your blood sugar?**   - Daily - Weekly - Once a month - Every 3 months - Every 6 months - Annually - Occasionally - No answer - Other   - Other frequency of glucose testing: ____ |
| **30. Do you monitor your blood pressure values?** That is, do you measure your blood pressure?   - Yes - No [skip to Q32] - No answer [skip to Q32] |
| **31. How often do you measure your blood pressure?**   - Daily - Weekly - Once a month - Every 3 months - Every 6 months - Annually - Occasionally - No answer - Other   - Other frequency of blood pressure measurement: ____ |
| **32. Have you ever been hospitalized because of your diabetes?**   - Yes - No [skip to Q36] - Don't know/No answer |
| **33. How many hospitalizations have you had due to diabetes?:** _____ |
| **34. Do you remember the date of the last hospitalization?**   - Yes - No [skip to Q36] |
| **35. Year of last hospitalization**: yyyy |
| **36. Have you ever had a cardiovascular event?** (Stroke, Heart attack, Ischemic heart disease, Obstructive artery disease, Congestive heart failure)   - Yes - No [skip to Q42 if men, Q43 if women] - Don't know/No answer [skip to Q42 if men, Q43 if women] |
| **37. How many cardiovascular events have you had?** |
| **38. Do you remember when?** If more than one cardiovascular event (year of the last episode):   - Yes - No [skip to Q42 if men, Q43 if women] |
| **39. Year of last cardiovascular event**: yyyy |
| **40. Do you remember the year it happened for the first time?**   - Yes - No [skip to Q42 if men, Q43 if women] |
| **41.** In case you've had more than one cardiovascular event:  **Date of the first cardiovascular event:** yyyy |
| **42.** [Only men] **Have you been diagnosed with erectile dysfunction or do you have impotence or problems having sexual relations since being diagnosed with diabetes?**   - Yes - No - Don't know/No answer |
| **43. Have you had a part of your foot or leg amputated because of diabetes?**   - Yes - No [skip to Q48] - No answer [skip to Q48] |
| **44. What was amputated?** ____ (If refused to answer: 999) |
| **45. Do you remember the date(s) of the amputations?**   - Yes - No [skip to Q48] |
| **46. Date of first (or only) amputation:** yyyy |
| **47. Date of second amputation:** yyyy |
| **Access to health services** |
| **48. Do you know your cardiovascular risk?**   - Yes - No - Don't know/No answer |
| **49. Have you been informed of your risk due to diabetes?**   - Yes - No - Don't know/No answer |
| **50. Have you had your kidneys checked since being diagnosed with diabetes?**   - Yes - No [skip to Q55] - Don't know/No answer [skip to Q55] |
| **51. Does your doctor explain the results of your blood or urine tests, indicating the state of your kidneys?**   - Yes - No, even though I get blood or urine tests - I don't get blood or urine tests - Don't know/No answer |
| **52. How often do you check your kidneys since the diabetes diagnosis?**   - Monthly - Every 3 months - Every 6 months - Annually - Occasionally - No answer - Other frequency than the previous ones   - Other kidney check-up frequency: ____ |
| **53. Do you remember the last time you were told about the state of your kidneys?**   - Yes - No [skip to Q55] |
| **54. Year of the last time you were told about your kidneys' condition:** yyyy |
| **55. Since being diagnosed with diabetes, have you seen an eye doctor to check your eyes?**   - Yes, for check-ups - Yes, because I have vision problems or had issues before the diabetes diagnosis - No [skip to Q59] - Don't know/No answer [skip to Q59] |
| **56.** [Only if YES in Q55] **Do you remember being diagnosed with anything?**   - Yes - No [skip to Q59] - Don't know/No answer [skip to Q59] |
| **57. What were you diagnosed with?:** ____ |
| **58. How many times have you had a retinal exam since being diagnosed with diabetes? : ______** (If unknown: 777 / If refused to answer: 999) |
| **59. Have you visited the dentist since being diagnosed with diabetes?**   - Yes, for check-ups - Yes, because I have pain or another problem with my teeth - No [skip to Q63] - Don't know/No answer [skip to Q63] |
| **60. How often do you visit the dentist?**   - Every 3 months - Every 6 months - Annually - Occasionally - Other   - Other dental visit frequency: ____ |
| **61. Do you remember being diagnosed with anything?**   - Yes - No [skip to Q63] - Don't know/No answer [skip to Q63] |
| **62. What were you diagnosed with?:** ____ |
| **63. Have you visited a psychologist since being diagnosed with diabetes?**   - Yes - No - Don't know/No answer |
| **64. Have you visited a nutritionist since being diagnosed with diabetes?**   - Yes - No - Don't know/No answer |
| **Participant's physical measurements and some lifestyle habits** |
| **65. How much do you weigh in kg?:** _____ (If unknown: 777 / If refused to answer: 999) |
| **66. How tall are you in meters?:** _____ (If unknown: 777 / If refused to answer: 999) |
| **67. Are you a smoker?**   - Yes, more than 10 cigarettes a day - Yes, less than 10 cigarettes a day - Yes, occasionally - Yes, I smoke another type of tobacco (regardless of frequency) - No - Refused to answer |
| **68. Do you drink alcoholic beverages such as beer, wine, spirits, rum, or guarapo?**   - Never - Rarely - 1 or more times a week - Every day - Refused to answer |
| **69. Do you exercise or engage in physical activity to improve your health? For example, walking, swimming, etc.**   - Yes - No - Refused to answer |
| **70. What activity?: ____**  Example: walking, swimming, dancing... |
| **Diabetes education** |
| The following questions refer to the information the patient has received about diabetes and advice on healthy lifestyle habits |
| **71. Have you ever been explained what diabetes is and the importance of controlling it yourself?**   - Yes - No - Don't know/No answer |
| **72. Have you ever been told about the benefits of having healthy habits? If so, which of the following:**  *Check as many options as apply.*   - Engage in physical activity - Maintain a healthy diet - Avoid alcohol - Don't smoke - I've never been told about these topics - Other advice   - Other advice received: _____ |
| **73. Have you ever been told about the existence and the possibility of joining a support group for chronic patients? (For example, diabetes and hypertension groups for: dance therapy, cooking, crafts, etc.)**   - Yes - No - Don't know/No answer |
| **74. Are you part of a support group?**   - Yes   - Which group is it? ____ - No - No answer |
| **Access to treatments** |
| **75. Has the doctor prescribed medications that you must take regularly for: Check as many options as necessary**   - Diabetes - Cholesterol - Hypertension - Does not need treatments, the doctor has not prescribed medications - Does not remember - No answer |
| **76. Does the subcentre or hospital provide you with the prescribed medications when you go for a check-up?**   - Yes, I always receive the medications without any issues [skip to Q80] - Yes, although sometimes they run out - No, I am asked to buy them at the pharmacy |
| **77. Regarding the lack of access to medications from the Health System, when does it occur?**   - Since the coronavirus pandemic started. - It has always happened, but now with the pandemic, it is more frequent. - Regardless, it frequently occurred even before the pandemic. - Other option different from the above, indicate which.   - Other frequency of lack of access to medications from the Health System: ____ |
| **78. What do you do when you do not have access to medications from the Health System? Check as many options as necessary**   - I stop taking them - I always buy them, I never run out of medications - I buy them, but despite this, I go through periods without taking the treatment - I take home remedies - I ask for help from an NGO - Other option different from the above, which one?: ______ - No answer |
| **79. How long did you go without taking your medications the longest time?** ______ |
| **80. Do you take home remedies to control your blood sugar, high blood pressure, cholesterol, or other health problems?**   - Yes - No [skip to Q82] - No answer [skip to Q82] |
| **81. Can you tell me which remedy you take?:** ____ |
| **Perceived Social Support – MSPSS** |
| **Family** |
| **82. I am confident that my family tries to help me.**   - Almost never - Sometimes - Often - Always or almost always - No answer |
| **83. My family gives me the help and emotional support I need.**   - Almost never - Sometimes - Often - Always or almost always - No answer |
| **84. I can talk to my family about my problems.**   - Almost never - Sometimes - Often - Always or almost always - No answer |
| **85. My family helps me make decisions.**   - Almost never - Sometimes - Often - Always or almost always - No answer |
| **Friends** |
| **86. I am confident that my friends try to help me.**   - Almost never - Sometimes - Often - Always or almost always - No answer |
| **87. I can count on my friends when I have problems.**   - Almost never - Sometimes - Often - Always or almost always - No answer |
| **88. When I have joys or sorrows, I can share them with my friends.**   - Almost never - Sometimes - Often - Always or almost always - No answer |
| **89. I can talk to my friends about my problems.**   - Almost never - Sometimes - Often - Always or almost always - No answer |
| **Other Significant People** |
| **90. When I need something, I know there is someone who can help me.**   - Almost never - Sometimes - Often - Always or almost always - No answer |
| **91. There is someone who offers me comfort when I need it.**   - Almost never - Sometimes - Often - Always or almost always - No answer |
| **92. When I have joys or sorrows, there is someone who can help me.**   - Almost never - Sometimes - Often - Always or almost always - No answer |
| **93. There is someone who cares about how I feel.**   - Almost never - Sometimes - Often - Always or almost always - No answer |
| **Health-Related Quality of Life - EuroQol-5D** |
| I will now ask you 5 questions, and you must select the option that best describes your health status TODAY |
| **94. Mobility:**   - I have no problems walking - I have some problems walking - I am confined to bed |
| **95. Self-care and grooming:**   - I have no problems with self-care and grooming - I have some problems washing or dressing - I cannot wash or dress myself |
| **96. Daily activities:** Example: working, studying, housework, family activities, or leisure activities.   - I have no problems performing my daily activities - I have some problems performing my daily activities - I cannot perform my daily activities |
| **97. Pain/Discomfort:**   - I have no pain or discomfort - I have moderate pain or discomfort - I have severe pain or discomfort |
| **98. Anxiety/Depression:**   - I do not feel anxious or depressed - I feel somewhat (moderately) anxious or depressed - I feel very anxious or depressed |
