# Supplementary material for: Characteristics associated with optimal blood sugar in individuals living with type 2 diabetes in hard-to-reach rural communities: results of a cross-sectional study in Esmeraldas, Ecuador
Source: BMC Public Health. 2025 Mar 25;25:1133. doi: 10.1186/s12889-025-22324-z (PMC11934518; doi:10.1186/s12889-025-22324-z)
Supplement: Supplementary file 2 — Supplementary Material 2. [file 12889_2025_22324_MOESM2_ESM.docx]

**Internal Consistency Analysis**

Cronbach’s alpha was used to evaluate the internal consistency of the MSPSS questionnaire. This metric assesses the degree of correlation between items within the same scale, with values ranging from 0 to 1. Higher values indicate better internal consistency, as follows:

- **Excellent:** >0.90
- **Good:** 0.80-0.90
- **Acceptable:** 0.70-0.80
- **Questionable:** 0.60-0.70
- **Poor:** <0.60

For the overall MSPSS questionnaire, Cronbach’s alpha was **0.91** (95% CI: 0.91–0.92), indicating excellent internal consistency.

The corrected item-total correlations ranged from 0.53 to 0.80 (Table S1), demonstrating that all items were positively correlated with the total scale and contributed meaningfully to the overall consistency. Removing any individual item did not improve the global Cronbach’s alpha, which remained at 0.91.

Table X. Corrected item-total correlations of the MSPSS questionnaire.

| **Item** | **Corrected Item-Total Correlation** | **Alpha if Item Removed** |
| --- | --- | --- |
| P3 | 0.53 | 0.91 |
| P4 | 0.56 | 0.91 |
| P8 | 0.66 | 0.90 |
| P11 | 0.63 | 0.90 |
| P6 | 0.70 | 0.90 |
| P7 | 0.68 | 0.90 |
| P9 | 0.73 | 0.90 |
| P12 | 0.70 | 0.90 |
| P1 | 0.76 | 0.90 |
| P5 | 0.79 | 0.90 |
| P2 | 0.80 | 0.90 |
| P10 | 0.77 | 0.90 |

Cronbach’s alpha was also calculated for the three MSPSS subscales:

- **Family:** (P3, P4, P8, P11) α = 0.88 (95% CI: 0.88–0.91)
- **Friends:** (P6, P7, P9, P12) α = 0.93 (95% CI: 0.92–0.94)
- **Significant Others:** (P1, P5, P2, P10) α = 0.95 (95% CI: 0.94–0.95)

These results confirm high internal consistency across all subscales, supporting their reliability as distinct components of the MSPSS.

**Confirmatory Factor Analysis (CFA)**

CFA was conducted to validate the proposed three-factor structure of the MSPSS. The analysis used the WLSMV (Diagonally Weighted Least Squares) estimator, appropriate for categorical variables such as Likert-type responses. Fit indices were computed to assess model adequacy.

**Three-Factor Model**

The three-factor model, based on prior research, assigned items as follows:

- Family: (P3, P4, P8, P11)
- Friends: (P6, P7, P9, P12)
- Significant Others: (P1, P5, P2, P10)

Fit indices for the three-factor model were:

- **Chi-Square (χ²):** 424.726, df = 51, p < 0.001
- **TLI (Tucker–Lewis Index):** 0.998
- **CFI (Comparative Fit Index):** 0.999
- **RMSEA (Root Mean Square Error of Approximation):** 0.119

While the chi-square statistic was significant, likely due to the large sample size, the TLI and CFI values were excellent, supporting the model. The RMSEA value was slightly elevated but within an acceptable range for categorical data.

**Alternative Models**

Two alternative models were tested for comparison:

1. **One-Factor Model** (all items grouped together):
   - **Chi-Square (χ²):** 21,088.588, df = 54, p < 0.001
   - **TLI:** 0.912
   - **CFI:** 0.928
   - **RMSEA:** 0.867
   - This model performed poorly, with a high RMSEA indicating poor fit.
2. **Two-Factor Model** (Family + Friends/Significant Others combined):
   - **Chi-Square (χ²):** 2,422.115, df = 53, p < 0.001
   - **TLI:** 0.990
   - **CFI:** 0.992
   - **RMSEA:** 0.294
   - Although this model performed better than the one-factor model, it still exhibited poorer fit compared to the three-factor structure.

**Conclusion**

The three-factor model demonstrated superior fit, aligning well with the theoretical framework of the MSPSS. The TLI and CFI values confirm its robustness, and the elevated RMSEA is attributable to the categorical nature of the data. These findings validate the appropriateness of the three-factor structure in explaining the relationships between the items and their latent constructs.
