# Supplementary material for: Characteristics associated with optimal blood sugar in individuals living with type 2 diabetes in hard-to-reach rural communities: results of a cross-sectional study in Esmeraldas, Ecuador
Source: BMC Public Health. 2025 Mar 25;25:1133. doi: 10.1186/s12889-025-22324-z (PMC11934518; doi:10.1186/s12889-025-22324-z)
Supplement: Supplementary file 3 — Supplementary Material 3. [file 12889_2025_22324_MOESM3_ESM.docx]

Supplementary File 3

For the construction of the logistic model for each subgroup (S1, S2, S4), variables were included if they met a p-value threshold of <0.10 and retained in the final model if they maintained a p-value of <0.05. Only the variables that remained significant in the final analysis are presented in the following tables.

**Table S1.** Characteristics associated with Optimal Fasting Capillary Blood Glucose in women (N=333)

|  | **OR** | **95% CI** | **p-value^†^** | **adjusted OR**** | **95% CI** | **p-value^†^** |
| --- | --- | --- | --- | --- | --- | --- |
| **Setting** |  |  |  |  |  |  |
| Rural | 1 |  |  | 1 |  |  |
| Urbanised | 2.35 | 1.25-4.41 | **0.008** | 2.33 | 1.23-4.42 | **0.010** |
| **Family support*** |  |  |  |  |  |  |
| Low | 1 |  |  | 1 |  |  |
| High | 0.44 | 0.20-0.98 | **0.045** | 0.48 | 0.22-1.08 | 0.076 |

*4 missing social support (MSPSS scale) ^†^p value marked in bold when <0.05 (Chi-squared test) **OR adjusted for variables (setting and family support) with sample size of 329 due to 4 missing values in family support.

**Table S2.** Characteristics associated with Optimal Fasting Capillary Blood Glucose in men (N=141)

|  | **OR** | **95% CI** | **p-value^†^** | **adjusted OR*** | **95% CI** | **p-value^†^** |
| --- | --- | --- | --- | --- | --- | --- |
| **Age** | 1.06 | 1.03-1.10 | **<0.001** | 1.04 | 1.00-1.08 | **0.045** |
| **Employment status** |  |  |  |  |  |  |
| Unemployed | 1 |  |  | 1 |  |  |
| Employed | 0.26 | 0.12-0.57 | **0.001** | 0.46 | 0.18-1.15 | 0.098 |
| **Education level** |  |  |  |  |  |  |
| No formal schooling | 1 |  |  | 1 |  |  |
| Primary school | 0.60 | 0.23-1.56 | 0.294 | 1.03 | 0.35-2.98 | 0.964 |
| Secondary school or higher education | 0.19 | 0.05-0.73 | **0.016** | 0.54 | 0.15-1.98 | 0.354 |

^†^p value marked in bold when <0.05 (Chi-squared test) *OR adjusted for variables (age, employment status and education level).

**Table S3.** Prevalence of optimal Fasting Capillary Blood Glucose result according to sociodemographic and clinical characteristics in individuals ≥40 years old (N=444).

|  | **Total population** | | | **Population ≥40 years olds** | | |
| --- | --- | --- | --- | --- | --- | --- |
| **Variables** | **N (%) optimal** | **Total** | **p-value^‡^** | **N (%) optimal** | **Total** | **p-value**^‡^ |
| **Sex** |  |  | **<0.001** |  |  | **<0.001** |
| Women | 46 (13.8) | 333 |  | 39 (12.7) | 308 |  |
| Men | 40 (28.4) | 141 |  | 40 (29.4) | 136 |  |
| **Age** (mean ±SD) | (64±15) | (60±13) | **<0.001** | (67±13) | (62±11) | **<0.001** |
| **Ethnic group*** |  |  | 0.453 |  |  | 0.353 |
| Mestizo | 22 (15.7%) | 140 |  | 21 (15.6%) | 135 |  |
| Afro | 58 (18.8%) | 308 |  | 52 (18.3%) | 285 |  |
| Indigenous | 6 (25.0%) | 24 |  | 6 (27.3%) | 22 |  |
| **Setting** |  |  | **0.032** |  |  | 0.148 |
| Rural | 48 (15.3%) | 313 |  | 47 (15.9%) | 296 |  |
| Urbanised | 38 (23.6%) | 161 |  | 32 (21.6%) | 148 |  |
| **Education level** |  |  | 0.144 |  |  | 0.249 |
| No formal schooling | 22 (25.3%) | 87 |  | 20 (24.1%) | 83 |  |
| Primary school | 48 (17.3%) | 278 |  | 44 (16.5%) | 266 |  |
| Secondary or higher | 16 (14.7%) | 109 |  | 15 (15.8%) | 95 |  |
| **Marital status** |  |  | 0.429 |  |  | 0.414 |
| Unpartnered | 28 (20.6%) | 136 |  | 26 (20.2%) | 129 |  |
| Partnered | 58 (17.2%) | 338 |  | 53 (16.8%) | 315 |  |
| **Employment status** |  |  | 1.000 |  |  | 0.795 |
| Unemployed | 57 (18.3%) | 312 |  | 51 (17.5%) | 292 |  |
| Employed | 29 (17.9%) | 162 |  | 28 (18.4%) | 152 |  |
| **Household earnings**^†^ |  |  | 0.214 |  |  | 0.443 |
| $100 or less | 49 (20.7%) | 237 |  | 43 (19.4%) | 222 |  |
| More than $100 | 31 (15.7%) | 198 | 0.942 | 31 (16.4%) | 189 |  |
| **T2DM duration in years, median (IQR)** | 7 (5-13) | 7 (5-11) | 0.938 | 7 (5-14) | 7 (5-11) | 0.680 |
| **Free T2DM treatment provided by the health centre** (self-reported)^β^ |  |  | 0.621 |  |  | 0.609 |
| Sometimes/never | 42 (16.3) | 257 |  | 39 (16.2) | 241 |  |
| Always | 38 (18.5) | 206 |  | 35 (18.1) | 193 |  |
| **Social support**^§^ |  |  | 0.536 |  |  | 0.799 |
| Low | 56 (19.1%) | 293 |  | 49 (18.2%) | 269 |  |
| High | 29 (16.5%) | 176 |  | 29 (17.1%) | 170 |  |
| **Family support**^§^ |  |  | **0.025** |  |  | 0.103 |
| Low | 16 (29.6%) | 54 |  | 12 (26.7%) | 45 |  |
| High | 69 (16.6%) | 415 |  | 66 (16.8%) | 394 |  |
| **Friend support**^§^ |  |  | 1.000 |  |  | 0.779 |
| Low | 62 (18.1) | 343 |  | 56 (17.5%) | 321 |  |
| High | 23 (18.3) | 126 |  | 22 (18.6%) | 118 |  |
| **Other significant support**^§^ |  |  | 0.700 |  |  | 1.000 |
| Low | 60 (18.7) | 321 |  | 53 (18.0%) | 295 |  |
| High | 25 (16.9) | 148 |  | 25 (17.4%) | 144 |  |
| **TOTAL** | **86 (18.1%)** | **474** |  | **79 (17.8%)** | **444** |  |

*2 missing †39 not reported ‡7 missing β11 missing §5 missing social support values (MSPSS scale) ^‡^p value marked in bold when <0.1 (t-student for age and Fisher’s exact test for the rest)

**Table S4.** Patients (≥40 years old) characteristics associated with an optimal Fasting Capillary Blood Glucose result (N=444)

|  | **OR** | **95% CI** | **p-value^†^** | **adjusted OR**** | **95% CI** | **p-value^†^** |
| --- | --- | --- | --- | --- | --- | --- |
| **Sex** |  |  |  |  |  |  |
| Women | 1 |  |  | 1 |  |  |
| Men | 2.87 | 1.75-4.73 | **<0.001** | 2.91 | 1.73-4.90 | **<0.001** |
| **Age** | 1.05 | 1.03-1.07 | **<0.001** | 1.05 | 1.03-1.07 | **<0.001** |
| **Family support*** |  |  |  |  |  |  |
| Low | 1 |  |  | 1 |  |  |
| High | 0.55 | 0.27-1.13 | 0.103 | 0.40 | 0.19-0.86 | **0.019** |

*5 missing social support (MSPSS scale) ^†^p value marked in bold when <0.05 (Chi-squared test) **OR adjusted for variables (sex and family support) with sample size of 439 due to 5 missing values in family support.
